# Ginger extract diminishes chronic fructose consumption-induced kidney injury through suppression of renal overexpression of proinflammatory cytokines in rats

**DOI:** 10.1186/1472-6882-14-174

**Published:** 2014-05-27

**Authors:** Ming Yang, Changjin Liu, Jian Jiang, Guowei Zuo, Xuemei Lin, Johji Yamahara, Jianwei Wang, Yuhao Li

**Affiliations:** 1 Faculty of Basic Medical Sciences, Chongqing Medical University, Chongqing 400016, China; 2 College of Laboratory Medicine, Chongqing Medical University, Chongqing 400016, China; 3 Endocrinology and Metabolism Group, Sydney Institute of Health Sciences/Sydney Institute of Traditional Chinese Medicine, Sydney, NSW 2000, Australia; 4 Pharmafood Institute, Kyoto 602-8136, Japan; 5 Department of Traditional Chinese Medicine, Chongqing Medical University, Chongqing 400016, China

**Keywords:** *Zingiber officinale* Roscoe, Renoprotection, Anti-inflammation, Metabolic abnormalities

## Abstract

**Background:**

The metabolic syndrome is associated with an increased risk of development and progression of chronic kidney disease. Renal inflammation is well known to play an important role in the initiation and progression of tubulointerstitial injury of the kidneys. Ginger, one of the most commonly used spices and medicinal plants, has been demonstrated to improve diet-induced metabolic abnormalities. However, the efficacy of ginger on the metabolic syndrome-associated kidney injury remains unknown. This study aimed to investigate the impact of ginger on fructose consumption-induced adverse effects in the kidneys.

**Methods:**

The fructose control rats were treated with 10% fructose in drinking water over 5 weeks. The fructose consumption in ginger-treated rats was adjusted to match that of fructose control group. The ethanolic extract of ginger was co-administered (once daily by oral gavage). The indexes of lipid and glucose homeostasis were determined enzymatically, by ELISA and/or histologically. Gene expression was analyzed by Real-Time PCR.

**Results:**

In addition to improve hyperinsulinemia and hypertriglyceridemia, supplement with ginger extract (50 mg/kg) attenuated liquid fructose-induced kidney injury as characterized by focal cast formation, slough and dilation of tubular epithelial cells in the cortex of the kidneys in rats. Furthermore, ginger also diminished excessive renal interstitial collagen deposit. By Real-Time PCR, renal gene expression profiles revealed that ginger suppressed fructose-stimulated monocyte chemoattractant protein-1 and its receptor chemokine (C-C motif) receptor-2. In accord, overexpression of two important macrophage accumulation markers CD68 and F4/80 was downregulated. Moreover, overexpressed tumor necrosis factor-alpha, interleukin-6, transforming growth factor-beta1 and plasminogen activator inhibitor (PAI)-1 were downregulated. Ginger treatment also restored the downregulated ratio of urokinase-type plasminogen activator to PAI-1.

**Conclusions:**

The present results suggest that ginger supplement diminishes fructose-induced kidney injury through suppression of renal overexpression of macrophage-associated proinflammatory cytokines in rats. Our findings provide evidence supporting the protective effect of ginger on the metabolic syndrome-associated kidney injury.

## Background

The metabolic syndrome is a well-established risk factor for diabetes, cardiovascular disease and mortality. Recently, studies have suggested that the metabolic syndrome may also contribute to the development of chronic kidney disease. Data from the Third National Health and Nutrition Examination Survey has shown an independent association between the metabolic syndrome and chronic kidney disease [1]. This connection has been further corroborated by the finding that the metabolic syndrome increases the risk of developing new-onset chronic kidney disease [2]. Indeed, renal injury is often seen in various animal models of the metabolic syndrome, such as Zucker diabetic fatty rats [3,4] and *db/db* mice [5].

The Western-style diet, characterized by an overavailability of food, with high intakes of high-fat foods, high-sugar desserts and drinks, as well as high intakes of red meat, refined grains, and high-fat dairy products, affects multiple metabolic functions and has been associated with a higher incidence of the metabolic syndrome. It has been suggested that the Western-style diet is a major risk factor for impaired kidney function and chronic kidney disease [6]. Notably, fructose has now become a major constituent of our modern diet. Fructose consumption has steadily increased over the past 30 years in parallel to the growth of the obesity/metabolic syndrome epidemic, and fructose and high-fructose corn syrup are ingredients in many commercially produced food products [1]. It has been hypothesized that fructose consumption in our diet may be among the factors that contribute to the epidemic of the metabolic syndrome and, consequently, to the epidemic of chronic renal disease [1,7-11]. This hypothesis is supported by the preliminary evidence demonstrating that high fructose consumption induces kidney damages in both rats [12-14] and mice [15].

Ginger (*Zingiber officinale* Roscoe, Zingiberacae) is one of the most commonly used spices and medicinal plants around the world. It has been demonstrated that ginger has pleiotropic pharmacological activities, such as gastrointestinal, analgesic, anti-inflammatory, antioxidant and cardiovascular activities [16,17]. The renoprotective effects of ginger have also been reported in the animal models of ischemia/reperfusion- [18,19], alcohol- [20,21], streptozotocin- [22] and carbon tetrachloride- [23] induced renal injuries. However, the efficacy of ginger on the metabolic syndrome-associated kidney damages remains unknown. We have recently demonstrated that ginger supplement improves fructose consumption-induced fatty liver [24] and adipose tissue insulin resistance [25] in rats. In the present study, we examined the impact of ginger on chronic fructose consumption-induced kidney injury in rats. Furthermore, the underlying mechanisms were also investigated.

## Methods

### Preparation and identification of the ethanolic extract of ginger

Ginger rhizomes were collected from the suburban area of Hanoi, Vietnam, and identified botanically by Professor Johji Yamahara, who is an expert in taxonomy. A voucher specimen was deposited in Pharmafood Institute, Kyoto, Japan (Voucher specimen No: PS0088). The extract used in the present study was prepared using an ethanolic method described previously [24]. Briefly, 5 kg of sliced dry ginger rhizomes including the skins were immersed in 5 L of 95% ethanol with intermittent shaking for 24 h, and then refluxed for 3 h by heating. The filtrate was evaporated below 45°C under reduced pressure. The residue (yield: 9.6%) was designated as an alcoholic extract. The extract was quantified by a HPLC method described previously [26] to contain two representative components: 6-gingerol and 6-shogaol at 4.4% and 1.1%, respectively.

### Animals, diet and experimental protocol

All animal procedures were in accordance with the ‘Principles of laboratory animal care’ (http://grants1.nih.gov/grants/olaw/references/phspol.htm) and were approved by the Animal Ethics Committee of Chongqing Medical University, China.

Male Sprague–Dawley rats aged 7–9 weeks (210–230 g) and standard laboratory chow were supplied by the Laboratory Animal Center, Chongqing Medical University, China. Rats were housed in a temperature controlled facility (21 ± 1°C, 55 ± 5% relative humidity) with a 12-h light/dark cycle. Animals were allowed free access to water and standard chow for at least 1 week prior to starting the experiments.

Research has shown that sugar-sweetened nonalcoholic beverages, such as soft drinks, appear as the major source of fructose for all classes of age considered, except for children younger than 6 years and adults older than 50 years [27]. Therefore, fructose in drinking water was used in the present study, in accordance to this rationale and the previous research protocol [24,25,28,29].

Dosage selection is of exceptional importance for pharmacological intervention. Excessively high dosages in animals may result in non-specific (“artificial”) effects, which may be dissociated with those in humans. A 35-day toxicity study in rats has demonstrated that the dried ginger powder at the dosages of 500, 1000 and 2000 mg/kg (equivalent to 48–192 mg/kg ethanolic extract used in the present study) was not associated with any mortalities and abnormalities in general conditions, behavior, growth, food and water consumption, hematological and blood biochemical parameters [26]. Previous studies have reported that treatment with dried ginger powder at a dosage of 200 [30] or 500 [31] mg/kg (equivalent to 19.2 or 48 mg/kg ethanolic extract used in the present study) alleviated streptozotocin-induced the metabolic syndrome-associated or renal dysfunctions in rats. In humans, 3–9 g dried ginger (equivalent to 288–864 mg ethanolic extract used in the present study) is the officially accepted dosages (Version 1, 2010 Chinese Pharmacopoeia). Based on the above information, the dosages of 20 and 50 mg/kg ethanolic extract were selected for the present study.

Twenty-four rats were divided into 4 groups (n = 6 per group): (1) water control, free access to water; (2) fructose control, free access to 10% fructose solution (w/v, preparation every day); (3) fructose + ginger 20 mg/kg and (4) fructose + ginger 50 mg/kg. There was no difference in body weight between the groups before treatments commenced. Animals in ginger-treated groups were administered ginger extract at 20 and 50 mg/kg (suspended in 5% Gum Arabic solution, gavage once daily) for 5 weeks, respectively. The rats in the corresponding water- and fructose-control groups received vehicle (5% Gum Arabic) alone. All rats had free access to the standard chow. To avoid stress and maintain accurate monitoring of fructose intake, only 2 rats were housed in a cage at any given time. The consumed chow and fructose solution were measured per 2 rats daily and the intake of fructose was calculated. Initial experiments showed that when compared to the vehicle alone, ginger treatment significantly increased the intake of the 10% fructose water when the rats were given free access. In order to exclude the influence resulting from differences in fructose intake, fructose consumption in the groups treated with the ginger extracts were adjusted by regulating the concentration of fructose solution daily to match that of the fructose control group on the previous day.

At the end of week 4, the rats were fasted overnight before blood samples were collected by retroorbital venous puncture under ether anesthesia at 9:00–12:00 am for determination of plasma concentrations of total cholesterol (kit from Kexin Institute of Biotechnology, Shanghai, China), triglyceride (Triglyceride-E kit, Wako, Osaka, Japan), glucose (kit from Kexin Institute of Biotechnology, Shanghai, China) and insulin (kit from Morinaga Biochemical Industries, Tokyo, Japan). At the end of week 5, the rats were weighed and killed by prompt dislocation of the neck vertebra. Kidneys and epididymal fat tissues were collected and weighed. The ratio of kidney weight to body weight was calculated. Segments of kidney were flash frozen in liquid nitrogen and stored at -80°C for subsequent determination of lipid contents and gene expression.

### Histological examination of kidney

All slides were examined by two different researchers in a blinded manner. Morphometric quantification was assessed by microscopy (IX-81, Olympus Corporation, Tokyo, Japan) using a NIH ImageJ (version 1.43) analyzing system.

A portion of kidney was fixed with 10% formalin and embedded in paraffin. Three-micron thick sections were cut and stained with hematoxylin and eosin. The sections were imaged and cross-sectional areas were estimated in glomeruli that were cut transversely. The outer borders of the glomeruli were traced at 200 × magnification, and glomerular tuft area was measured. Fifty glomeruli per kidney were counted, and the mean values of these estimates were used in analyses. To further investigate the damage, an additional section (two-micron thickness) fixed in a 4% paraformaldehyde solution was stained with periodic acid-Schiff (PAS) and examined as previously described [13] using light microscopy and blinded assessors. Tubular size was determined by outlining each tubular profile. 200 tubules in each kidney section were examined. Tubular injury (by counting the number of tubules that exhibited cast formation, slough and dilation of tubular epithelial cells) was evaluated.

To determine the degree of collagen fiber accumulation, a kidney section (two-micron thickness) was stained with Masson’s trichrome. Forty fields in different sections were randomly selected, and Masson’s trichrome-stained area (blue) and total tissue area were determined. Their ratio was calculated as interstitial collagen deposit (fibrosis).

To observe lipid accumulation, six-micron frozen kidney sections were stained with Oil Red O.

### Determination of triglyceride and total cholesterol contents in kidney

Triglyceride and total cholesterol contents in kidney were determined as described previously [24,28]. Briefly, 100 mg of tissue was homogenized and extracted with 2 ml of isopropanol. After centrifugation (1000 × g, 10 min at 4°C), the triglyceride and total cholesterol contents in supernatants were determined enzymatically (Wako, Osaka, Japan).

### Real-time PCR

Total RNA was isolated from kidneys of individual rats using TRIzol (Takara, Dalian, China). cDNA was synthesized using M-MLV RTase cDNA Synthesis Kit (Takara, Dalian, China) according to the manufacturer’s instructions. Real-Time PCR was performed with the CFX 96 Real Time PCR Detection System (Biorad Laboratories Inc, Hercules, CA, USA) using the SYBR® Premix Ex Taq™ II (Takara, Dalian, China). The sequences of primers are shown in Table 1. The gene expression from each sample was analysed in duplicates and normalized against the internal control gene β-actin. Levels in water control rats were arbitrarily assigned a value of 1.

**Table 1:** Primer sequences for Real Time PCR assays

**Gene**	**Forward primers**	**Reverse primers**
β-actin	ACGGTCAGGTCATCACTATCG	GGCATAGAGGTCTTTACGGATG
MCP-1	CGGTTTCTCCCTTCTACTTCCTG	GCTCTGCCTCAGCCTTTTATTG
CCR-2	GAAGACCCAAAGACCAAGATGC	TCTGACAACAAAGCAGGAGGTG
CD68	ACTGGGGCTCTTGGAAACTACAC	CCTTGGTTTTGTTCGGGTTCA
F4/80	ATCGCTGCTGGCTGAATACG	GCAACCTCGTATCCTTGAGCTTAG
TNF-α	ATGGGCTCCCTCTCATCAGTTC	CTCCTCCGCTTGGTGGTTTG
IL-6	GTTGCCTTCTTGGGACTGATGT	GGTCTGTTGTGGGTGGTATCCT
TGF-β	GATCAGTCCCAAACGTCGAGG	CAGGTGTTGAGCCCTTTCCAG
PAI-1	GAACGCCCTCTATTTCAACGG	AGTTCCAGGATGTCGTACTCGTG
uPA	GCTTCGGACAAGAGAGTGCCA	GCCATAGTAGTGAGGCTGCTTGC

Sequences: 5’ to 3’.

### Data analysis

All results are expressed as means ± SEM. Data were analyzed by ANOVA using the StatView software (Version 5.0.1, SAS Institute Inc. USA), and followed by The Student-Newman-Keuls test to locate the differences between groups. *P* < 0.05 was considered to be statistically significant.

## Results

### General characteristics of the effects of ginger extract in fructose-fed rats

Compared to water drinking, intake of 10% fructose solution decreased intake of chow (Table 2). After 4-week supplementing with fructose, plasma concentrations of insulin, total cholesterol and triglyceride were elevated, whereas glucose concentration remained unchanged (Table 2). Rats in the fructose control and fructose ginger (20 and 50 mg/kg) groups showed similar intakes of fructose and chow. However, supplementing with a ginger extract at 50 mg/kg significantly decreased plasma concentrations of glucose, insulin and triglyceride, but it did not affect plasma total cholesterol concentration in fructose-fed rats (Table 2). Ginger extract at 20 mg/kg showed minimal effect across all parameters shown in Table 2.

**Table 2:** General parameters (n=6)

	**Water**	**Fructose**		
	**Ginger 0**	**Ginger 0**	**Ginger 20**	**Ginger 50**
Fructose intake (g/2 rats/5 w)	-	845±33	827±13	832±18
Chow intake (g/2 rats/5 w)	1993±100*	1083±74	1156±29	1156±52
Body weight (g)	333±7	338±11	324±7	318±6
Epididymal fat weight (g)	4.14±0.46	4.81±1.10	4.49±0.46	3.93±0.25
Plasma glucose (mmol/L)	4.74±0.11	4.92±0.23	4.87±0.25	4.02±0.18*
Plasma insulin (pmol/L)	8.57±1.10*	16.26±2.86	15.60±0.88	7.91±0.44*
Plasma TG (mmol/L)	0.47±0.03*	1.01±0.09	0.96±0.08	0.45±0.07*
Plasma TC (mmol/L)	1.83±0.06*	2.57±0.12	2.60±0.08	2.75±0.14
Plasma BUN (mmol/L)	6.78±0.64	7.10±1.04	7.71±0.86	7.85±0.57
Plasma creatinine (μmol/L)	45.81±6.12	51.92±9.23	44.21±7.62	45.03±4.64

TG, triglyceride; TC, total cholesterol; *vs* fructose control (ginger 0), *P<0.05.

### Effects on kidney-related variables in rats

**Figure 1:**
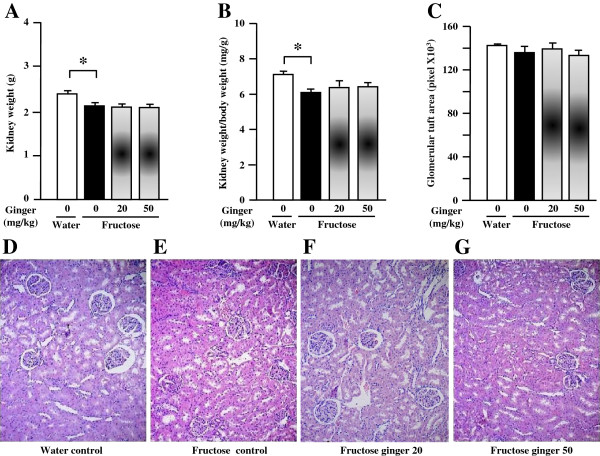
**Kidney weight (A), the ratios of kidney weight to body weight (B), glomerular tuft area (C) and representative images showing renal histology (hematoxylin and eosin, magnification: 100X, D-G) in rats.** The fructose control rats had free access to 10% fructose in their drinking water over 5 weeks, while the consumption of fructose in the ginger (20 or 50 mg/kg)-treated (by gavage daily) rats was adjusted to that of the fructose control rats. The water-control rats had free access to tap water. All values are means ± SEM (n = 6 each group). *P < 0.05.

**Figure 2:**
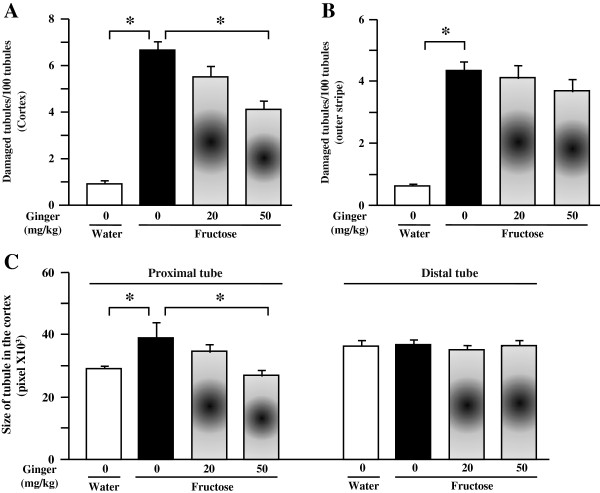
**Damaged tubules characterized by focal cast formation, slough and dilation of tubular epithelial cells in the cortex (A) and outer of stripe (B), and size of proximal and distal tubes in the cortex (C) of kidney in rats.** The fructose control rats had free access to 10% fructose in their drinking water over 5 weeks, while the consumption of fructose in the ginger (20 or 50 mg/kg)-treated (by gavage daily) rats was adjusted to that of the fructose control rats. The water-control rats had free access to tap water. All values are means ± SEM (n = 6 each group). *P < 0.05.

**Figure 3:**
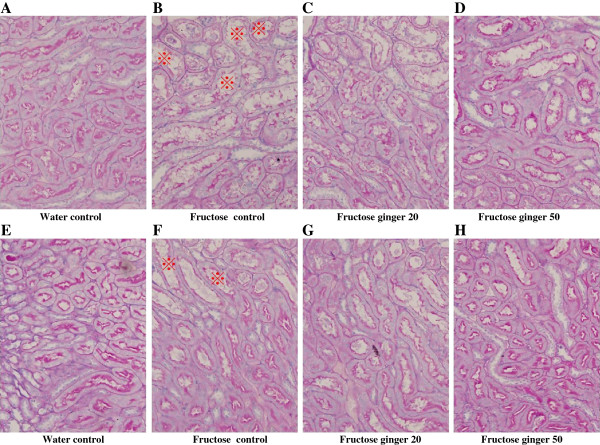
**Representative images showing tubular damages characterized by focal cast formation, slough and dilation of tubular epithelial cells (with ※, periodic acid-Schiff staining, magnification: 400X) in the cortex of kidney in rats (A-H).** The fructose control rats had free access to 10% fructose in their drinking water over 5 weeks, while the consumption of fructose in the ginger (20 or 50 mg/kg)-treated (by gavage daily) rats was adjusted to that of the fructose control rats. The water-control rats had free access to tap water.

**Figure 4:**
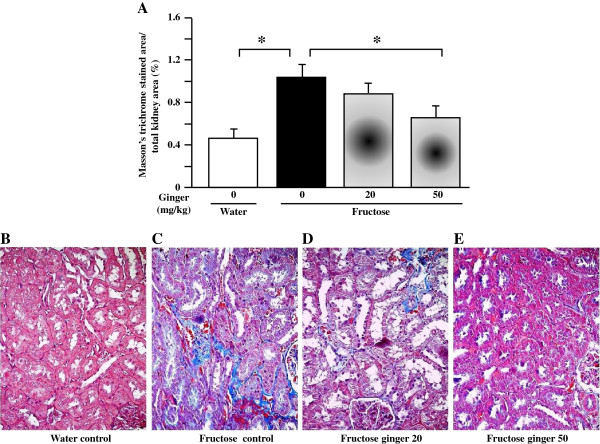
**The ratio of Masson’s trichrome-stained area to total tissue area in the renal interstitium (A) and representative images showing Masson’s trichrome-stained interstitial collagen deposit (Blue) (magnification: 400×) in the kidney of rats (B-E).** The fructose control rats had free access to 10% fructose in their drinking water over 5 weeks, while the consumption of fructose in the ginger (20 or 50 mg/kg)-treated (by gavage daily) rats was adjusted to that of the fructose control rats. The water-control rats had free access to tap water. All values are means ± SEM (n = 6 each group). *P < 0.05.

**Figure 5:**
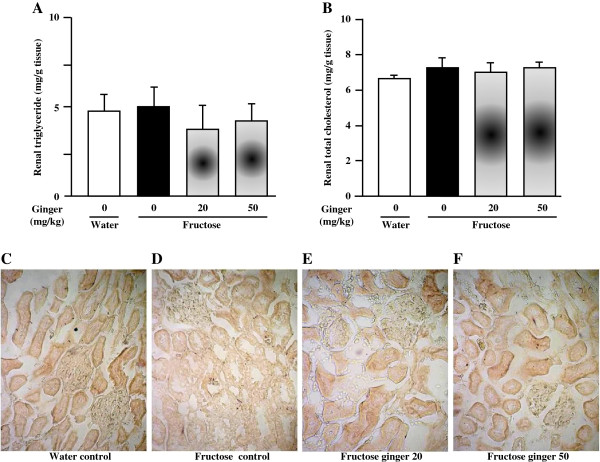
**Renal triglyceride (A) and total cholesterol (B) contents, and representative images showing histology of kidney (C-F, Oil Red O staining, 400X) in rats.** The fructose control rats had free access to 10% fructose in their drinking water over 5 weeks, while the consumption of fructose in the ginger (20 or 50 mg/kg)-treated (by gavage daily) rats was adjusted to that of the fructose control rats. The water-control rats had free access to tap water. All values are means ± SEM (n = 6 each group). *P < 0.05.

Fructose feeding did not significantly affect plasma BUN and creatinine (Table 2), body weight (Table 2) and glomerular tuft area (Figure 1C, D-G) in rats. However, it decreased kidney weight (Figure 1A) and the ratio of kidney weight to body weight (Figure 1B). Supplementing with a ginger extract at 20 and 50 mg/kg did not significantly affect these parameters in fructose-fed rats (Table 2, Figures 1A-G).Importantly, fructose induced a pronounced increase in tubular damage in both the cortex and outer stripe of the medullas characterized by the focal cast formation, slough and dilation of tubular epithelial cells (Figures 2A, B, 3A and B). Further analysis showed that fructose feeding increased the size of proximal, but not distal tubules in the cortex (Figure 2C, Figures 3A-H). Treatment with ginger extract at 50 mg/kg significantly decreased the damage of tubules in the cortex, but not in the outer stripe of the medullas (Figures 2A and B). Furthermore, this supplement decreased the enlargement of proximal tubules, whereas the size of distal tubules in the cortex was not affected (Figure 2C, Figures 3A-H). Ginger extract at 20 mg/kg failed to significantly affect these variables (Figures 2A-C, Figures 3B and C, and Figures 3F and G).In addition, fructose feeding increased the ratio of the Masson’s trichrome-stained area to total tissue area in the renal interstitium (Figures 4A-C). Supplementing with a ginger extract at 50 mg/kg significantly inhibited this increase (Figures 4A, C and E), whereas the lower dosage of ginger extract showed minimal effect (Figures 4A, C and D).In contrast to the tubular injury and interstitial fibrosis, renal triglyceride (Figure 5A) and total cholesterol (Figure 5B) contents were not altered by fructose feeding. Unchanged lipid accumulation was further confirmed by Oil Red O staining (Figures 5C and D). Treatment with a ginger extract at either low or high dosage did not affect renal lipid contents in fructose-fed rats (Figures 5A, B, E and F).

### Renal gene expression profiles in rats

**Figure 6:**
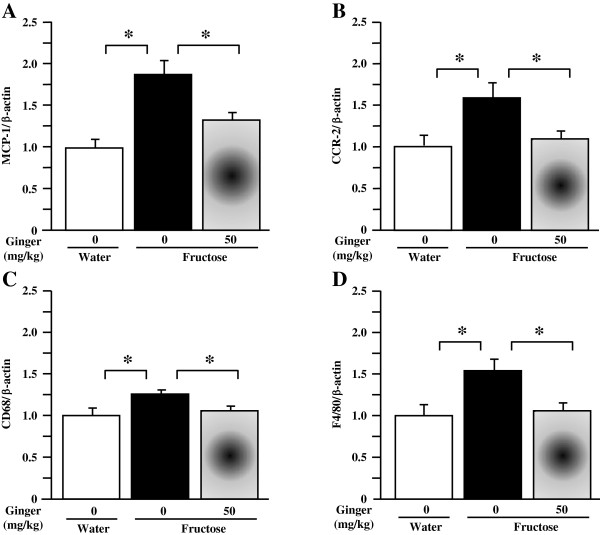
**Renal mRNA expression of monocyte chemotactic protein (MCP)-1 (A), chemokine (C-C motif) receptor (CCR)-2 (B), CD68 (C) and F4/80 (D) in rats.** The fructose control rats had free access to 10% fructose in their drinking water over 5 weeks, while the consumption of fructose in the ginger (20 or 50 mg/kg)-treated (by gavage daily) rats was adjusted to that of the fructose control rats. The water-control rats had free access to tap water. mRNA was determined by Real-Time PCR. Levels in water-control rats were assigned a value of 1. All values are means ± SEM (n = 6 each group). *P < 0.05.

**Figure 7:**
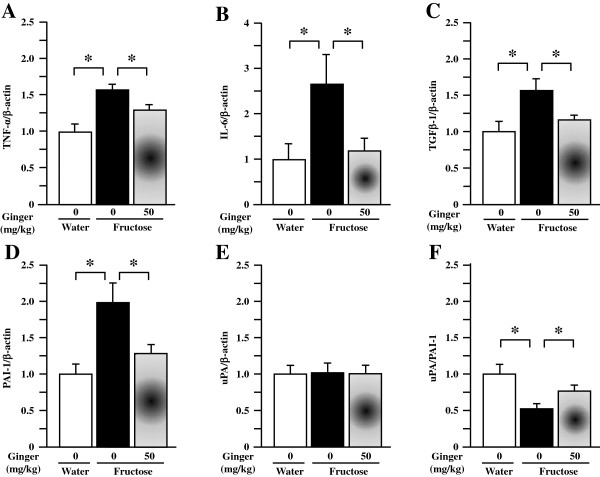
**Renal mRNA expression of tissue necrosis factor (TNF)-α (A), interleukin (IL)-6 (B), transforming growth factor (TGF)-β1 (C), plasminogen activator inhibitor (PAI)-1 (D), urokinase-type plasminogen activator (uPA) (E) and the ratio of uPA to PAI-1 (F) in rats.** The fructose control rats had free access to 10% fructose in their drinking water over 5 weeks, while the consumption of fructose in the ginger (20 or 50 mg/kg)-treated (by gavage daily) rats was adjusted to that of the fructose control rats. The water-control rats had free access to tap water. mRNA was determined by Real-Time PCR. Levels in water-control rats were assigned a value of 1. All values are means ± SEM (n = 6 each group). *P < 0.05.

As the supplement with ginger extract at 20 mg/kg showed negligible effects on all phenotypic parameters, comparisons in gene expression were restricted to water control, fructose control and fructose ginger 50 mg/kg groups.By real-time PCR, fructose feeding increased renal expression of mRNAs corresponding to monocyte chemotactic protein (MCP)-1 (Figure 6A), chemokine (C-C motif) receptor (CCR)-2 (Figure 6B), CD68 (Figure 6C), F4/80 (Figure 6D), TNF-α (Figure 7A), IL-6 (Figure 7B), transforming growth factor (TGF)-β1 (Figure 7C) and plasminogen activator inhibitor (PAI)-1 (Figure 7D). Although urokinase-type plasminogen activator (uPA) was not altered (Figure 7E), the ratio of uPA to PAI-1 expression was significantly downregulated by fructose feeding (Figure 7F). Ginger supplement substantially suppressed renal overexpression of MCP-1, CCR-2, CD68, F4/80, TNF-α, IL-6, TGF-β1 and PAI-1 (Figures 6A-D and Figures 7A-D), and restored the downregulated ratio of uPA to PAI-1 (Figure 7F).

## Discussion

Ginger has been demonstrated to protect rats from ischemia/reperfusion- [18,19], alcohol- [20,21], streptozotocin- [22] and carbon tetrachloride- [23] induced renal injuries. Recently, we have demonstrated that ginger supplement improves fructose consumption-induced fatty liver [24] and adipose tissue insulin resistance [25] in rats. The present study investigated the effects of ginger on chronic fructose consumption-associated kidney injury. Consistent with the previous findings [13], the present results demonstrate that five-week fructose consumption induced kidney remodeling as characterized by focal cast formation, slough and dilation of tubular epithelial cells in the cortex and outer stripe of the medullas, and excessive interstitial collagen deposit in rats. However, these pathological changes were accompanied by minimal alteration in glomerular structure and concentrations of BUN and plasma creatinine. It is possible that the mild initial histological changes do not induce pronounced alterations in renal functionality. Supplementing with a ginger extract (50 mg/kg) attenuated the proximal tubular damage and interstitial fibrosis in the kidneys and these effects were accompanied by improvements in hyperinsulinemia and hypertriglyceridemia. Therefore, these results present evidence suggesting that ginger possesses protective effect against the initial stages of the metabolic syndrome-associated kidney injury.

Renal inflammation is known to play an important role in the initiation and progression of tubulointerstitial injury in the kidneys [32,33]. Fructose has been demonstrated to induce production of macrophage-associated MCP-1 in human kidney proximal tubular cells [34]. Fructose consumption leads to cortical tubular damage with inflammatory infiltrates [12]. MCP-1 promotes monocyte and macrophage migration and activation, and upregulates expression of adhesion molecules and other proinflammatory cytokines. Studies indicate that the local expression of MCP-1 at sites of renal injury promotes macrophage adhesion and chemotaxis through ligation of CCR-2 [35]. In patients, tubular MCP-1 is elevated in progressive renal diseases [36] and albuminuria is associated with MCP-1 and macrophage infiltration [37]. The infiltrated macrophages produce numerous proinflammatory cytokines, such as TNF-α [38], which has been shown to mediate inflammation in several models of renal injury, including tubulointerstitial injury [39]. It has been reported that gingerols, [6]-shogaol and 1-dehydro-[10]-gingerdione inhibit lipopolysaccharide-stimulated release and gene expression of proinflammatory cytokines including MCP-1 and IL-6 in RAW 264.7 macrophages and cultured primary rat astrocytes [40-43]. In addition, another component of ginger, known as zingerone, has also been shown to suppress the inflammatory action of macrophages and release of MCP-1 from adipocytes, thereby blunting the inflammatory response of adipose tissue in obesity [44]. These findings have been corroborated by a study we have recently conducted in rats demonstrating the modulatory effects of ginger on adipose expression of macrophage-related proinflammatory cytokines thereby ameliorating fructose-induced adipose tissue insulin resistance [25]. The present study found that the ginger extract containing [6]-gingerol and [6]-shogaol was able to suppress fructose-induced overexpression of MCP-1, CCR-2, CD68 and F4/80 (two important macrophage markers [45,46]), TNF-α and IL-6 in the kidneys. These findings are consistent with the attenuation of proximal tubular injury. Thus, the renoprotective effect of ginger supplement is associated with suppression of renal overexpression of macrophage-associated proinflammatory cytokines.

Proinflammatory cytokines are associated with renal fibrosis. It has been demonstrated that blockading MCP-1 and its receptor CCR-2 pathway reduces renal fibrosis [47]. The activated macrophages also produce other proinflammatory cytokines, such as IL-6, TGF-β1 and PAI-1 [38]. IL-6 was shown to enhance TGF-β1 signaling *via* modulation of TGF-β1 receptor trafficking, an effect that may enhance renal fibrosis [48]. TGF-β1 may activate the plasmin system by stimulating gene expression of PAI-1, the principal inhibitor of plasminogen activation [49]. PAI-1 has a number of important roles in pathophysiological processes, such as inhibition of fibrinolysis, regulation of extracellular matrix turnover and activation of proenzymes and latent growth factors that promote tissue fibrosis and sclerosis [48]. In progressive renal diseases, PAI-1 has been identified as a critical mediator of glomerulosclerosis and interstitial fibrosis [50,51]. The altered uPA to PAI-1 ratio reflects a change from a profibrinolytic to an antifibrinolytic state [52]. The shift toward the uPA-enriched profibrinolytic state favors renal collagen degradation. Given its pathophysiological role, studies into TGF-β1 have found that [6]-gingerol inhibits its stimulation of myofibroblast differentiation and collagen production in nasal polyp-derived fibroblasts [53] and of proteoglycan core protein synthesis in human vascular smooth muscle cells [54]. In the present study, fructose-induced upregulation of MCP-1, CCR-2, IL-6, TGF-β1 and PAI-1 gene expression in kidney was suppressed by ginger supplement (50 mg/kg). The ratio of uPA to PAI-1 was also restored. Thus, ginger-elicited diminishment of renal interstitial fibrosis is also associated with suppression of renal overexpression of proinflammatory cytokines, thereby improving profibrinolytic state.

Lipid accumulation in nonadipose tissues has been increasingly recognized to contribute to organ injury through a process termed lipotoxicity. There is substantial evidence that excess renal lipids can cause injury in animal models of metabolic disease (obesity, metabolic syndrome and diabetes mellitus), chronic kidney disease, acute renal injury of several etiologies, as well as aging [55]. Lipotoxic cellular dysfunction and injury occur through several mechanisms such as release of proinflammatory and profibrotic factors [55]. Fructose consumption may induce excessive lipid accumulation in liver [27]. We have recently demonstrated that treatment with the ethanolic extract of ginger attenuates fructose-induced fatty liver in rats [24]. In the present study, however, five-week fructose feeding did not alter renal accumulation of triglyceride and total cholesterol in rats. Ginger treatment (20 and 50 mg/kg) also did not affect renal lipid contents in fructose-fed rats. Thus, it is unlikely that ginger treatment ameliorates fructose-induced renal injury in rats via modification of renal lipid metabolism.

While there are numerous constituents in ginger, the two prominent components [6]-gingerol and [6]-shogaol have been implicated in the majority of pharmacological activities associated with ginger [16]. At this point, further investigation is needed to broaden our collective knowledge regarding the details surrounding the therapeutic actions of ginger. Specifically, whether [6]-gingerol, [6]-shogaol, or a combination thereof is responsible for the diminishment of fructose-induced renal injury, their specific function on macrophages, and the manner in which they suppress proinflammatory cytokines.

## Conclusion

Our present results demonstrate that supplement with ginger extract at 50 mg/kg attenuates chronic fructose consumption-induced kidney injury in rats by suppressing renal overexpression of proinflammatory cytokines. Our findings provide evidence supporting the benefit of ginger supplement for the metabolic syndrome-associated kidney injury.

## Abbreviations

BUN: Blood urea nitrogen; BW: Body weight; CCR: Chemokine (C-C motif) receptor; IL: Interleukin; MCP: Monocyte chemoattractant protein; PAI: Plasminogen activator inhibitor; PAS: Periodic acid-schiff; TGF: Transforming growth factor; TNF: Tumor necrosis factor; uPA: Urokinase-type plasminogen activator.

## Competing interests

The authors have declared that no conflict of interest exists.

## Authors’ contribution

CL and MY performed the experiments, analyzed/interpreted data and drafted the manuscript. JJ, GZ, XL and JY analyzed/interpreted data. JW and YL contributed to the concept, designed experiments, analyzed/interpreted data and finalized the manuscript. All coauthors reviewed and discussed the paper. All authors read and approved the final manuscript.

## Pre-publication history

The pre-publication history for this paper can be accessed here:

http://www.biomedcentral.com/1472-6882/14/174/prepub
